# Association between the COVID-19 Vaccine and Preventive Behaviors: Panel Data Analysis from Japan

**DOI:** 10.3390/vaccines11040810

**Published:** 2023-04-06

**Authors:** Eiji Yamamura, Youki Kohsaka, Yoshiro Tsutsui, Fumio Ohtake

**Affiliations:** 1 Department of Economics, Seinan Gakuin University, Fukuoka 814-8511, Japan; 2 Department of Management Information, Kyoto College of Economics, Kyoto 610-1195, Japan; 3 Faculty of Social Relations, Kyoto Bunkyo University, Kyoto 611-0041, Japan; 4 Center for Infectious Disease Education and Research, Osaka University, Osaka 565-0871, Japan

**Keywords:** vaccine, COVID-19, preventive behaviors, norm, Japan, panel data

## Abstract

The coronavirus (COVID-19) vaccine is key to reducing the probability of contracting COVID-19. The vaccine is generally known to prevent severe illness, death, and hospitalization as a result of the disease and for considerably reduce COVID-19 infection risk. Accordingly, this might significantly change an individual’s perceived risk of altering everyday behaviors. For instance, the proliferation of vaccination is anticipated to reduce preventive behaviors such as staying at home, handwashing, and wearing a mask. We corresponded with the same individuals monthly for 18 months from March 2020 (early stage of COVID-19) to September 2021 in Japan to independently construct large sample panel data (N = 54,007), with a participation rate of 54.7%. We used a fixed effects model, controlling for key confounders, to determine whether vaccination was associated with a change in preventive behaviors. The major findings are as follows. Contrary to the prediction, (1) based on the whole sample, being vaccinated against COVID-19 led people to stay at home; however, it did not change the habit of handwashing and wearing a mask. Especially after the second shot, respondents were likelier to stay at home by 0.107 (95% CIs: 0.059–0.154) points on a 5-point scale compared to before the vaccination. Dividing the entire sample into young and old, (2) those aged ≤ 40 years were more likely to go out after being vaccinated, and (3) people over 40 years of age were more likely to stay at home (similar to the first result). Preventive behaviors impact all individuals during the current pandemic. Informal social norms motivate people to increase or maintain preventive behaviors even after being vaccinated in societies where these behaviors are not enforced.

## 1. Introduction

To reduce coronavirus (COVID-19) transmission, many countries implemented lockdowns. People were obliged to follow preventive behaviors enforced by their governments; otherwise, they were penalized [1]. Economic activities were suspended following restrictions on daily movements [2,3]. Subsequently, lockdowns significantly reduced the contact rate and spread of COVID-19 [4,5,6]. 

However, lockdown restrictions create significant economic losses [7,8] and negatively impact individuals’ mental health [9,10,11,12]. Therefore, the Japanese government has declared a “state of emergency” wherein preventive behaviors are strongly required but not enforced. Without enforcement, Japanese people continued to adhere to preventive behaviors by staying at home, washing their hands frequently, and wearing masks [13]. 

Various vaccines against COVID-19 have been developed and distributed worldwide. Vaccination is expected to significantly reduce the spread of COVID-19. The number of newly reported cases of COVID-19 has been observed to have reduced in countries where the vaccines were rapidly adopted [14]. Accordingly, the mental condition of vaccinated individuals improved [15,16,17].

In Japan, the first case of COVID-19 infection was confirmed on 16 January 2020. Based on the New Infectious Diseases Law, the Japanese Government recognized COVID-19 as a “designated infectious disease.” Accordingly, the Japanese government implemented enforced hospitalization and restricted work if a person was infected with COVID-19. However, the number of infected people surged in April 2020. To mitigate the rapid spread of COVID-19, the government declared a state of emergency on 7 April 2020. Yet, the government only requested citizens to wash their hands and wear masks and avoid direct contact and social gatherings. Unlike the lockdown adopted in other countries, they were neither punished nor penalized under the state of emergency, even if people did not follow the request. Inevitably, people could behave according to their free will, although moral and informal social norms deterred them slightly from practicing undesirable behaviors. On 25 May 2020, the state of emergency was deregulated as the number of daily infected persons remarkably reduced. Subsequently, until Autumn 2021, COVID-19 patients were cyclically observed; thus, the state of emergency was declared four times during the study period between March 2020 and September 2021. In Japan, vaccines were introduced in February 2021 [18]. Initially, vaccination was strictly limited to those who had a higher risk of contracting COVID-19. The regulation was then relaxed in stages, which led to more widespread vaccination.

Widespread vaccination can promote economic activities as vaccinated people may not exhibit rigid preventive behaviors and readopt their pre-COVID-19 lifestyle [19,20,21,22]. While Japanese people did not change their consumption behavior even after vaccination, they were likely to increase their consumption after the eradication of COVID-19 [23]. 

Hence, analyzing the mechanism behind the unexpected consumption behavior in Japan is crucial. Using monthly individual-level panel data, we investigate whether individuals’ preventive behaviors change before and after vaccination. Further, following the COVID-19 pandemic, individuals’ reactions to changes in policies related to COVID-19 differ according to their situation [13]. Time lags exist for the diffusion of vaccination among different generations in Japan. Our simulation study shows that determining economic loss during the pandemic depends on where COVID-19 is allocated according to age group [24]. Hence, we compared the difference in the impact of the uptake of the COVID-19 vaccine between the young and old generations, considering that they are exposed to different situations. Thanks to the risk reduction of COVID-19 infection, vaccinated people become less likely to engage in preventive behaviors than before being vaccinated in China [25] and Bangladesh [26]. Further, studies did not find that vaccinated people decreased preventive behaviors in comparison to those who were not vaccinated in China [27] and the UK [28]. The influence of vaccination on preventive behaviors might differ according to the type of behavior. The aim of this study is to compare the effect of the vaccination on various preventive behaviors.

## 2. Materials and Methods

### 2.1. Data Collection

We commissioned the research company INTAGE to conduct an internet survey based on their experience and reliability. The first wave of queries was conducted from 13 March 2020 to 16 March 2020, recording 4359 observations with a participation rate of 54.7%. Participants registered with INTAGE were recruited for this study. The sampling method was designed to gather a representative sample of the Japanese population in terms of gender, age, educational background, and residential area. Basically, the overall sample is designed to be representative of the Japanese adult population, and the responding sample is weighted to be representative of the Japanese population. However, the sample population was restricted to ages 16–79; individuals aged 15 years and below were considered too young to be registered with INTAGE, and we considered individuals over 80 years of age too old to answer pertinent questions. To construct a sample representative of the Japanese population, INTAGE recruited participants for a survey from among pre-registered people. Participants were randomly selected to fill the pre-specified quotas. While INTAGE provided monetary incentives to participants upon study completion, the company did not provide specific information regarding said incentives.

Japanese citizens aged 16–79 years were selected for the survey. Internet surveys were conducted repeatedly for 15 separate times (“waves”) almost every month with the same individuals to construct the panel data. However, the survey could not be conducted for the period between July 2020 and September 2020 due to a shortage of research funds. We resumed the surveys after receiving additional funds from October 2020. 

Respondents from the first wave were targeted in the subsequent waves to record how the same respondent changed their perceptions and behaviors during the COVID-19 pandemic. During the study period, some of the respondents stopped taking the surveys, while others did not take the surveys at all. The total number of observations used in this study was 54,007.

### 2.2. Ethical Considerations

Our study was performed according to the relevant guidelines and regulations. The ethics committee of Osaka University approved all survey procedures, and informed consent was obtained from all participants. The ethics approval number of Osaka University for this study is R021014.

After being informed about the purpose of the study and their right to quit at any time, participants agreed to participate. The completion of the entire questionnaire was considered to indicate the participants’ consent.

### 2.3. Measurements

The survey questionnaire contained basic questions about demographics such as age, gender, and educational background. Fifteen waves were conducted between March 2020 and September 2021. As the main variables, the respondents were asked questions concerning preventive behaviors as follows: 

“Within a week, to what degree have you practiced the following behaviors? Please answer based on a scale of 1 (I have not practiced this behavior at all) to 5 (I have completely practiced this behavior).”

(1) Staying indoors.
(2) Not going out to the workplace (or school).
(3) Not going out to events or travel.
(4) Washing my hands thoroughly.
(5) Wearing a mask.

The answers to these questions served as proxies for the following variables for preventive behaviors: staying indoors, not going out for work, not participating in leisure activities outside the home, frequently handwashing, and wearing masks. Larger values indicate that respondents are more likely to engage in preventive behaviors. Staying indoors generally captures the degree of staying (not going out) at home. For more specific behaviors, we asked about the type of voluntary restraint while going out. Not going out for work captures the degree of avoidance of going out for work or school. Not participating in leisure activities outside the home captures the degree of avoidance of going out for events or travel. In the case of the former, preventive behavior depends on the condition of the workplace or school. Hence, respondents may have been obliged to go to work or school. The latter is more likely to depend on an individual’s decision-making. Further, we asked about the subjective probability of contracting COVID-19 and their perception of the severity of COVID-19.

### 2.4. Methods

We used panel data, pooling cross-sections across time. Based on the panel data, we used a fixed effects (FE) model regression to control for time-invariant individual fixed effects. The FE model is a type of linear regression model widely used in economics. The estimation result using an FE model is equivalent to the results of a linear regression model with dummies of individuals who are frequently included in each period [29,30,31]. The FE model can be applied to linear regression and also to other types of regression, such as conditional logistic regression [32]. In the case of this study, 4358 dummies are included to control for individuals’ characteristics that do not change during the period, such as gender, educational background, childhood experience, and so on. Hence, 4358 cofounders are included, and they reflect differences between individuals. Even if various time-variant cofounders are included, unobserved individual characteristics cannot be captured. This inevitably results in omitted variable biases [29,31]. In the FE model, an individual’s time-invariant characteristics can be completely controlled, reducing the possibility of omitted variable biases. However, the estimated results for time-invariant confounders cannot be obtained. For instance, we cannot calculate how educational background is correlated with the dependent variable. 

Furthermore, in the model used in this study, we also controlled for differences between different time points of the survey. Government policy and the degree of spread of COVID-19 vary in the 15 time points in the survey. Similar to time-invariant individual effects, controlling for differences between time points is impossible, even if we include various cofounders that vary according to time points (e.g., macroeconomic shocks and policy effects). We should control for this to mitigate omitted variable biases. Therefore, we included 14 dummies for different waves, and we set the first wave as the reference group. Thus, the common time-specific effects covering all parts of Japan are completely controlled. Meanwhile, the effect of government policy cannot be calculated, which was implemented throughout Japan simultaneously.

Therefore, in this model, we controlled for not only unobservable individual effects but also unobservable time effects. This type of FE model is specifically called the two-way error component regression model [31]. Estimation results are less likely to suffer from omitted variable biases in this model; however, we cannot calculate the estimated effects of various policies, educational backgrounds, and gender differences. In other words, this study focuses on the correlation between vaccination and preventive behaviors. The statistical software used in this study was Stata/MP 15.0. 

The estimated function of FE model takes the following form:

*Y*_it_ = α_1_ *VACCINE FIRST*_it_ + α_2_ *VACCINE SECOND_1*_it_ + α_3_ *VACCINE SECOND_2*_it_ + α_4_ *VACCINE SECOND_3*_it_ + α_5_ *VACCINE SECOND_4*_it_ + α_6_ *PROB COVID19*_it_ + α_7_ *SEVERITY COVID19* _it_ +α_8_ *EMERGENCY* _it_ + k_t_ + m_i_ + u_itg_,

In this formula, *Y_itp_* represents the dependent variable for individual i and wave t. Y includes preventive behaviors captured by the five proxy variables defined in Table 1: *STAYING INDOORS, NOT GOING TO WORK, NOT FOR LEISURE, HANDWASHING*, and *WEARING MASKS*. These are discrete ordered variables from 1 to 5. Larger values of these variables can be interpreted as meaning that the respondents are more likely to exhibit preventive behavior. In the same specification, we conduct five estimations separately, and the regression parameters are denoted as α. The error term is denoted by *u*, and *k_t_* represents the effects of different time points that were controlled by 14 wave dummies, where the first wave is the reference group. Various shocks occurred simultaneously throughout Japan at each time point. Wave dummies were included to control for this. The estimation method is the fixed effects (FE) model, and the time-invariant individual-level fixed effects are represented by m_i_. This means that the model controls various individual characteristics that do not change, even if time has passed. Hence, sex, educational background, and various factors were controlled for. During the study period, respondents’ ages increased by only one year, and the timing of this change depended on their birthdays. Therefore, the variation in age reflects birthdays in the FE model. Therefore, age was not included in the model, even though the results did not change after including age in the model. A simple FE linear regression model was used in this study. However, proxy variables for preventive behaviors include the scores of the scale, which are ordered from 1 to 5. Therefore, these are discrete variables and not continuous ones. For closer examination, we use the FE-ordered logit model to conduct the estimation because the dependent variable is a multinomial-ordered response [33,34].

Key independent variables are vaccination dummies; the vaccine first controls the effect of the first shot. Various pharmaceutical companies have developed vaccines. However, the Japanese government approved only those developed by Pfizer and BioNTech. People are obliged to get the second shot within a month of taking the first shot to make the vaccine effective. This rule applied to the Pfizer and BioNTech vaccines. The effect of the second shot should then be estimated separately. Further, we investigated how the association between the vaccine and preventive behaviors changes over time. Therefore, we included *VACCINE SECOND_1, VACCINE SECOND_2, VACCINE SECOND_3,* and *VACCINE SECOND_4.*

In Japan, declarations of a state of emergency significantly affected behaviors [13,35]. However, the timing of the declarations differed by area. Therefore, the effect of the declaration could not be captured by wave dummies. Hence, we include *EMERGENCY* to control for the effects of the declaration of emergency. Furthermore, the subjective perception of COVID-19 is expected to influence preventive behavior. For instance, people are more likely to be cautious about COVID-19 if they consider the probability of contracting COVID-19 and the severity of the damage done by COVID-19 as higher [36,37]. To control for this, we include *PROB COVID19* and *SEVERITY COVID19*. Although their results were not reported, we also controlled for the following factors: the number of persons infected with COVID-19 and the deaths caused by COVID-19 in residential areas at each time point. Subjective emotions, such as anxiety, fear, and anger, are also controlled by including *ANGER, FEAR*, and *ANXIETY*. 

The motivation for obtaining a vaccination shot depends on age [38,39]. The association between vaccination and preventive behaviors varies according to an individual’s situation. Moreover, considering the whole sample, we conduct estimations by dividing it into two: young (below 40 years) and old (equivalent or over 40 years). 

## 3. Results

As explained in the previous section, the sampling method was designed to gather a representative sample of the Japanese population in terms of gender, age, educational background, and residential area. Our survey included Japanese citizens between 16 and 79 years of age from all regions of Japan and covered all parts of Japan. The age range and demographic composition of the sample were almost equivalent to those of the 2015 Japan Census (Figure S1 in Supplementary Materials). However, some gaps exist in the aged population between the census data and the survey data used in this study. The percentage of younger participants in the survey sample was lower. Particularly, the gap is large for the age group 16–20, reflecting that they are too young to be registered with INTAGE. The percentage of participants belonging to the age groups 66–70 and age group 71–75 in our sample is higher than that in the census, whereas those belonging to the age group 76–79 in our sample are lower. On the one hand, retired older people have enough spare time to participate in the survey. On the other hand, older people were not young enough to participate in the questionnaire. Figure S2 (in Supplementary Materials) demonstrates the composition of the population in 47 residential prefectures using our survey sample and census sample. Figure S2 shows a similar geographical population distribution. Overall, the sample used in the present study can be considered representative of public opinion in Japan.

In the first survey, a questionnaire was sent to 7968 people who were registered with a research company, and its participation rate was 54.7%. Therefore, 4359 observations were obtained. Subsequently, in every survey, the questionnaire was sent to respondents who participated in the first survey; thus, 4359 people. Table 2 shows the number of observations and loss rates, as some respondents did not respond. The loss rate is approximately 20% in each survey. Table 3 indicates mean and s.d of variables used in this study. In this study, we used the unbalanced panel data, including when some respondents did not participate in several surveys. Table 4 indicates ercentage of those who took the COVID-19 vaccine. As shown later, estimation results were exhibited in Table 5, Table 6, Table 7, Table 8, Table 9 and Table 10. The estimation results were almost the same even when we used the balanced panel data where all respondents participated in all surveys. Its estimation results corresponding to Table 6 were reported in Supplementary File S2.

Table 3 suggests that the mean values of staying indoors and not going out for work were 2.91 and 2.94, respectively. Meanwhile, the value for not participating in leisure activities outside the home was 4.12. This means that people are more likely to go to work or school than to engage in leisure activities. This suggests that events or travel are considered less essential than work or school. Staying indoors consists of both essential and non-essential components. Overall, not going out to work is critical in determining the probability of staying indoors. Not going out for work is determined not by an individual’s will but by instruction from the workplace or school. Similar to refraining from leisure activities, the mean values of handwashing and wearing masks were slightly larger than 4. This is because handwashing and wearing masks are likely to depend on an individual’s willingness. 

We also asked respondents whether they had taken the first shot of the vaccine against COVID-19 and whether they had completed the second vaccine shot. In Japan, vaccination began on February 2021 [18]. During this period, the first group eligible for the shot was strictly limited to health workers before the inoculation program was expanded to include the general public. Vaccination for older people aged 65 and over has been implemented since April 2021, and 75% of older people have been vaccinated as of July 2021 [40]. Additionally, the government has begun implementing COVID-19 vaccination programs at workplaces and campuses where workers and students can get vaccinations since June [41]. 

**Table 3:** Definitions of key variables and their basic statistics.

Variables	Mean	s.d.
*STAYING I* *NDOORS*	2.91	1.25
*NOT GOING TO WORK*	2.94	1.73
*NOT FOR LEISURE*	4.12	1.18
*HANDWASHING*	4.14	0.95
*WEARING MASK*	4.41	1.05
*VACCINE FIRST*	0.03	0.17
*VACCINE SECOND*	0.06	0.24
*VACCINE SECOND_1*	0.03	0.18
*VACCINE SECOND_2*	0.02	0.14
*VACCINE SECOND_3*	0.01	0.08
*VACCINE SECOND_4*	0.001	0.04
*PROB_COVID19*	20.4	22.3
*SEVERITY COVID19*	3.57	1.21
*EMERGENCY*	0.29	0.45
*FEAR*	3.06	1.14
*ANXIETY*	3.28	1.15
*ANGER*	2.98	1.10
*AGE*	48.7	17.3
*MALE*	0.50	0.50
*UNIVERSITY*	0.43	0.49

The 10th wave survey was conducted directly after February 2021. In the sample used in this study, respondents who received the shot appeared from the 12th wave conducted in May 2021. On the dummies for vaccination, the mean values of *VACCINE SECOND_1, VACCINE SECOND_2, VACCINE SECOND_3*, and *VACCINE SECOND_4* are 0.03, 0.02, 0.01, and 0.001, respectively. Therefore, in the entire sample, people who received the second shot at the time of the survey accounted for 3%. The number of people who received the second shot last month, 2 months ago, and 3 months ago were 2%, 1%, and only 0.1%, respectively. The entire sample covered first-eleventh waves, where nobody received the shot, so percentages were very low. The vaccine was distributed to healthcare workers first, followed by older people, and then others. Therefore, the percentage declines with people who received the second shot earlier.

To check the change in vaccination rate, Table 4 shows the percentage of vaccinated people in each wave. Contrary to the vaccination dummy, Table 4 indicates the aggregated values containing both the first and second vaccinated people regardless of the vaccination time point. Therefore, the percentage of vaccinated individuals is expected to increase over time. Consistent with this inference, Table 4 indicates that the percentage of vaccinated people rapidly increased from 8.2% in May 2021 to 64.2% at the beginning of September in the sample. This rate is similar to that of 65.2% in September using country-wide data [42]. In the subsample of people over 40, the rate increased from 9.1% in May 2021 to 72.3%, almost twice as high as that in the subsample of younger people in each wave. Thus, the data in this study are representative of the actual situation in Japan.

**Table 4:** Percentage of those who took the COVID-19 vaccine.

Waves	Dates	All %	Age > 40 %	Age ≤ 40 %
1	13–16 March 2020	0	0	0
2	27–30 March 2020	0	0	0
3	10–13 April 2020	0	0	0
4	8–11 May 2020	0	0	0
5	12–15 June 2020	0	0	0
6	23–28 October 2020	0	0	0
7	4–8 December 2020	0	0	0
8	15–19 January 2021	0	0	0
9	17–22 February 2021	0	0	0
10	24–29 March 2021	0	0	0
11	23–26 April 2021	0	0	0
12	28–31 May 2021	8.2	9.1	5.4
13	25–30 June 2021	25.1	30.7	7.8
14	30 July–4 August 2021	50.0	58.3	23.8.
15	27 August–1 September 2021	64.2	72.3	39.5

Note: We did not distinguish between respondents who took only the first shot and those who took the second shot.

**Figure 1:**
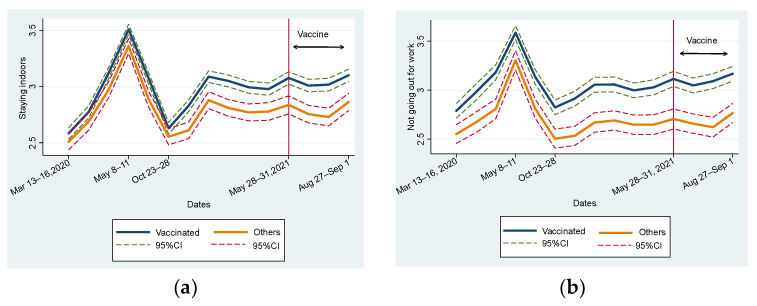

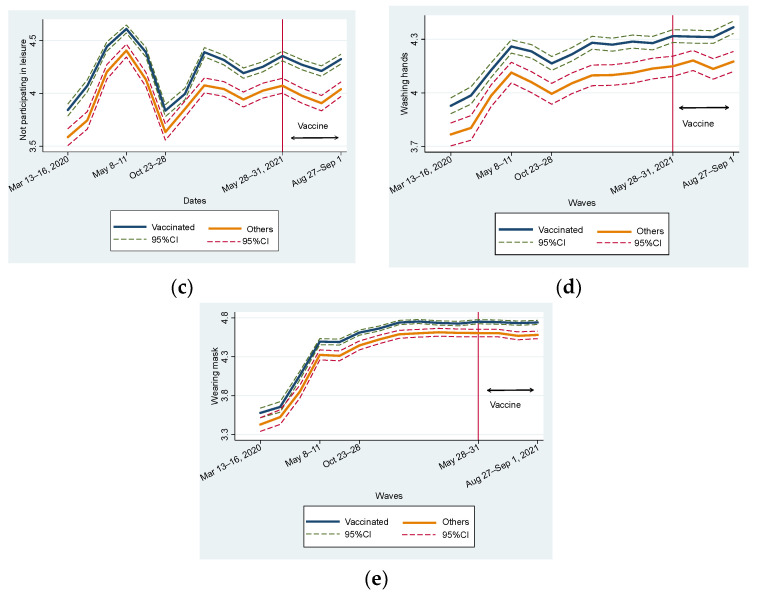
(**a**) Change in staying indoors. (**b**) Change in not going out for work. (**c**) Change in leisure activities. (**d**) Change in handwashing. (**e**) Change in wearing masks.

Figure 1 illustrates the change in five preventive behaviors from the first to the fifteenth waves by dividing the sample into vaccinated and unvaccinated groups. Figure 1 covers the periods before and after vaccination. Therefore, nobody was vaccinated from the first to the eleventh waves, and the left part of the vertical line is shown in Figure 1. In this study, people vaccinated during any period were included in the vaccinated group. Furthermore, we did not distinguish people who received the second shot from those who only received the first. For instance, one who received their first shot in the fifteenth wave was included in the vaccinated group. Thus, Figure 1 indicates how people who did not intend to be vaccinated behaved differently from vaccinated people during the period when the vaccine was not distributed.

Figure 1a indicates that the vaccinated group was more likely to stay at home than the unvaccinated group throughout the study period. The trends of both groups were similar. At the first declaration of a state of emergency in all parts of Japan from the third to fourth waves, people immediately complied and stayed at home. After calling the first declaration, the level of staying at home declined to the level before the declaration. Later, the state of emergency was declared and consecutively called off four times. In response to this, the level of staying at home increased but did not peak during the first declaration. This level was more stable in 2021 than in 2020. However, we should note that the gap in behavior increased, especially after the eighth wave and after entering 2021. Similar tendencies were observed in Figure 1b,c for “not going out for work” and “not participating in leisure activities outside the home.”

As shown in Figure 1d,e, in terms of changes in handwashing and wearing masks, similar to Figure 1a–c, the vaccinated group showed consistently higher levels of adherence than the unvaccinated group. However, the gap in handwashing was larger than that in wearing masks. Mask-wearing behavior is motivated by self-regarding risk preferences and other-regarding concerns [38,43,44,45,46]. In other words, the effect of interpersonal interaction possibly reduces the gap in wearing masks.

Compared to Figure 1a–c, a remarkable difference exists in the trends shown in Figure 1d,e. The level of handwashing and wearing masks almost constantly rose, indicating that people became more inclined to wash their hands and wear a mask even after the declaration of a state of emergency. This is consistent with the fact that many people began habitually washing their hands in response to the 2009 influenza pandemic, and their habits have persisted over the years [47].

Overall, we did not observe the effect of vaccination by comparing the time periods before and after the distribution of the COVID-19 vaccine (Figure 1). Figure 1 presents a change in mean values; thus, various factors that influence preventive behaviors are not controlled. We then examined the fixed effects regression model to closely examine the relationship between vaccination and preventive behaviors. Before scrutinizing the difference in effects between the first and second vaccinations, Table 5 shows the simple mean difference test before and after vaccination. We limited the sample to those who had been vaccinated during the study period. Further, we divided the sample into subsamples before the first vaccination and subsample after it. For a rough comparison, we did not distinguish between the first and second vaccinations. Table 3 shows that all types of preventive behaviors show larger values after vaccination than before vaccination. Further, these differences were statistically significant at the 1% level. We carefully considered the difference between before and after vaccination (DIF) for the following: *STAYING INDOORS* (DIF 0.14 [95% CI: 0.10–0.18]), *NOT GOING TO WORK* (DIF 0.19 [95% CI: 0.13–0.24]), *NOT FOR LEISURE* (DIF 0.10 [95% CI: 0.07–0.14]), *HANDWASHING* (DIF 0.16 [95% CI: 0.13–0.19]), and *WEARING MASK* (DIF 0.29 [95% CI: 0.26–0.32]). This implies that people are more likely to engage in preventive behaviors after than before vaccination.

**Table 5:** Mean difference test before and after vaccination using a sample of respondents who were vaccinated during the studied period.

Dates	Before (1)	After (2)	Difference (2)–(1)
*STAYING I* *NDOORS*	2.96	3.11	0.14 *** (0.10–0.18)
*NOT GOING TO WORK*	3.03	3.22	0.19 *** (0.13–0.24)
*NOT FOR LEISURE*	4.21	4.32	0.10 *** (0.07–0.14)
*HANDWASHING*	4.19	4.36	0.16 *** (0.13–0.19)
*WEARING MASK*	4.45	4.75	0.29 *** (0.26–0.32)

Note: Numbers within parentheses are 95% CI. *** *p* < 0.01.

### 3.1. Full Sample Estimations 

The coefficient of confounders indicates marginal effects (ME). Table 6 presents the estimation results of the FE model using the entire sample. We begin by examining key variables of vaccination dummies. Except for Column (5), where the WEARING MASK is the dependent variable, the coefficients of the vaccination dummies show a positive sign in most cases. *VACCINE FIRST* is statistically significant only in columns (1) and (4), and its statistical significance is not at the 1% level. Furthermore, *VACCINE SECOND_1* and *VACCINE SECOND_2* are statistically significant at the 1% level in most cases in columns (1)–(3); in contrast, *VACCINE SECOND_3* and *VACCINE SECOND_4* are not significant in any column. Furthermore, the effects of *VACCINE SECOND_1* are (ME 0.099 [95% CI: 0.058–0.140]), (ME 0.070 [95% CI: 0.007–0.132]), and (ME 0.077 [95% CI: 0.031–0.122]), in columns (1), (2), and (3), respectively. Thus, compared with unvaccinated people, vaccinated people are more likely to stay at home by 0.099 points, not to go to work by 0.070 points, and not to go out for leisure by 0.077 points on a 5-point scale. This indicates that the degree of staying home increased by 1.98%, not going to work by 1.40 %, and not going out for leisure by 1.54% directly after the second shot than before vaccination. Turning to *VACCINE SECOND_2,* its effects increased (ME 0.123 [95% CI: 0.061–0.187]), (ME 0.123 [95% CI: 0.042–0.204]), and (ME 0.106 [95% CI: 0.047–0.165]), in columns (1), (2), and (3), respectively. Through conversion, this indicates that the degree of staying home and not going to work increased by 2.46% and not going out for leisure by 2.12% two months after the second shot than before vaccination.

**Table 6:** FE model. Dependent variables are preventive behaviors.

	(1) *STAYING INDOORS*	(2) *NOT GOING TO WORK*	(3) *NOT FOR LEISURE*	(4) *HANDWASHING*	(5) *WEARING MASK*
*VACCINE FIRST*	0.057 ** (0.007–0.106)	0.032 (−0.016–0.080)	0.027 (−0.014–0.069)	0.026 * (−0.001–0.054)	−0.001 (−0.047–0.045)
*VACCINE SECOND_1*	0.099 *** (0.058–0.140)	0.070 ** (0.007–0.132)	0.077 *** (0.031–0.122)	0.006 (−0.029–0.041)	−0.006 (−0.046–0.034)
*VACCINE SECOND_2*	0.123 *** (0.061–0.187)	0.123 *** (0.042–0.204)	0.106 *** (0.047–0.165)	−0.012 (−0.051–0.027)	−0.0003 (−0.048–0.047)
*VACCINE SECOND_3*	0.097 (−0.023–0.217)	0.092 (−0.020–0.206)	0.018 (−0.097–0.133)	0.035 (−0.023–0.095)	−0.027 (−0.094–0.040)
*VACCINE SECOND_4*	0.014 (−0.335–0.365)	−0.019 (−0.167–0.128)	−0.106 (−0.341–0.128)	−0.018 (−0.142–0.105)	−0.040 (−0.177–0.095)
*PROBABILITY COVID19*	−0.291 (−1.064–0.480)	−0.532 (−1.657–0.591)	0.103 (−0.328–0.535)	0.428 * (−0.079–0.936)	−0.472 (−1.208–0.263)
*SEVERITY COVID19*	0.016 *** (0.007–0.026)	0.017 * (−0.001–0.036)	0.036 *** (0.019–0.053)	0.018 *** (0.006–0.030)	0.033 *** (0.021–0.045)
*EMERGENCY*	0.022 (−0.008–0.054)	0.034 ** (0.007–0.062)	0.047 *** (0.017–0.078)	−0.001 (−0.016–0.014)	0.013 (−0.002–0.020)
*ANGER*	0.035 *** (0.025–0.046)	0.023 *** (0.007–0.039)	0.054 *** (0.038–0.069)	0.018 *** (0.009–0.027)	0.009 (−0.002–0.020)
*FEAR*	0.051 *** (0.035–0.066)	0.031 *** (0.009–0.053)	0.045 *** (0.024–0.067)	0.018 *** (0.007–0.028)	0.036 *** (0.021–0.052)
*ANXIETY*	0.037 *** (0.023–0.051)	0.021 * (−0.001–0.044)	0.049 *** (0.034–0.063)	0.026 *** (0.015–0.036)	0.029 *** (0.012–0.047)
*WAVE 1*			<Default>		
*WAVE 2*	0.126 *** (0.085–0.167)	0.092 *** (0.048–0.137)	0.170 *** (0.113–0.226)	0.043 ** (0.009–0.077)	0.047 *** (0.016–0.079)
*WAVE 3*	0.446 *** (0.355–0.536)	0.273 *** (0.181–0.365)	0.516 *** (0.450–0.582)	0.177 *** (0.144–0.210)	0.386 *** (0.332–0.440)
*WAVE 4*	0.829 *** (0.738–0.920)	0.687 *** (0.592–0.782)	0.698 *** (0.622–0.773)	0.329 *** (0.286–0.373)	0.833 *** (0.755–0.915)
*WAVE 5*	0.435 *** (0.353–0.517)	0.269 *** (0.185–0.354)	0.517 *** (0.456–0.577)	0.289 *** (0.260–0.318)	0.862 *** (0.802–0.924)
*WAVE 6*	0.052 (−0.014–0.119)	−0.010 (−0.082–0.060)	0.025 (−0.038–0.090)	0.237 *** (0.203–0.272)	1.010 (0.942–1.079)
*WAVE 7*	0.161 *** (0.101–0.221)	0.017 (−0.046–0.081)	0.157 *** (0.108–0.206)	0.267 *** (0.235–0.300)	1.061 *** (0.993–1.130)
*WAVE 8*	0.389 *** (0.307–0.470)	0.141 *** (0.061–0.220)	0.458 *** (0.391–0.525)	0.317 *** (0.278–0.356)	1.122 *** (1.040–1.204)
*WAVE 9*	0.368 *** (0.306–0.431)	0.153 *** (0.095–0.210)	0.417 *** (0.0357–0.477)	0.319 *** (0.274–0.365)	1.146 *** (1.068–1.224)
*WAVE 10*	0.344 *** (0.271–0.416)	0.140 *** (0.070–0.210)	0.323 *** (0.259–0.387)	0.339 *** (0.305–0.374)	1.133 *** (1.065–1.201)
*WAVE 11*	0.304 *** (0.239–0.369)	0.132 *** (0.071–0.192)	0.373 *** (0.309–0.436)	0.336 *** (0.300–0.372)	1.126 *** (1.053–1.198)
*WAVE 12*	0.375 *** (0.304–0.446)	0.195 *** (0.132–0.259)	0.442 *** (0.375–0509)	0.363 *** (0.322–0.405)	1.139 *** (1.064–1.213)
*WAVE 13*	0.309 *** (0.233–0.385)	0.141 *** (0.079–0.201)	0.365 *** (0.206–0.350)	0.372 *** (0.334–0.410)	1.132 *** (1.059–1.206)
*WAVE 14*	0.282 *** (0.207–0.357)	0.156 *** (0.078–0.232)	0.278 *** (0.206–0.350)	0.355 *** (0.315–0.395)	1.111 *** (1.036–1.185)
*WAVE 15*	0.346 *** (0.248–0.444)	0.231 *** (0.141–0.322)	0.384 *** (0.299–0.469)	0.413 *** (0.360–0.467)	1.135 *** (1.051–1.219)
Adj R^2^ Obs.	0.37 54,007	0.65 54,007	0.37 54,007	0.62 54,007	0.49 54,007

Note: Numbers within parentheses are 95% CI. For convenience, the coefficient of PROBABILITY COVID19 is multiplied by 1000. Although the effects are not reported, the model includes the number of deaths and infected persons in residential prefectures at the times of surveys as control variables and serves as proxies for mental conditions such as fear, anxiety, and anger. *** *p* < 0.01, ** *p* < 0.05, * *p* < 0.10.

Overall, these imply that people who have completed their second shot choose to stay at home and not go out for work, school, or leisure. This tendency was observed in the month when they received the second shot and the following month. Particularly, the effect was larger in subsequent months. However, this effect was resolved. 

Before estimation, we hypothesize that getting vaccinated might encourage people to go out more as COVID-19 is less likely to have a detrimental effect on vaccinated people. Our findings contradict this hypothesis. After vaccination, some side effects are normal and expected, including pain, swelling, redness at the injection site, chills, mild fever, tiredness, headaches, joint pain, or muscle ache [48]. This may reduce the incentive to go out. However, side effects resolve within a few days; side effects may affect one’s ability to perform daily activities for a few days [48]. However, experiencing side effects does not explain the increase in staying at home in the following months.

In our interpretation, social norms for promoting preventive behaviors were formed through the experience of COVID-19. According to an expert, “After being vaccinated, it is important you continue the behaviors that protect yourself and others against COVID-19… This is because COVID-19 vaccines have proven effective at stopping people from developing the virus, but we do not yet know whether they prevent people from passing the infection onto others.” [48]. This instruction is considered a “nudge” to influence behavior [49,50,51]. Social media exposure to COVID-19 information influences the adoption of preventive attitudes and behaviors by shaping risk perception [52]. Arguably, this kind of instruction after vaccination contributes to forming social norms through the media.

People would usually perceive having done something wrong when they go against social norms. Alternatively, vaccinated people will likely be punished if they break the norm. Especially at the early stage of vaccine distribution, the vaccine supply was low, so only healthcare workers and adults could receive shots. Furthermore, making reservations for vaccination was quite challenging. Hence, the number of highly advantaged vaccinated individuals was very small. They would be seriously criticized if they broke the norm. If vaccinated people understood the inference, they still refrained from going out.

Hence, the norms become more effective for vaccinated people as they are less likely to obey them. The gap in preventive behaviors between vaccinated and unvaccinated individuals returned to pre-vaccination levels but did not decrease despite two or three months having passed. On handwashing and wearing masks, the dummies for vaccination did not show any significant negative signs. Therefore, vaccination did not hamper people’s adherence to preventive behaviors.

The model specification shows that subjective perception about COVID-19 is controlled by *PROB COVID19* and *SEVERITY COVID19*. Particularly, the coefficient of *SEVERITY COVID19* exhibits a positive sign and is statistically significant at the 1% level in all estimations. This is consistent with the inference that people are more likely to exhibit preventive behaviors if they consider the damage done by COVID-19 to be larger. 

In most cases, wave dummies presented a positive sign and statistical significance at the 1% level, except *WAVE 6*. This suggests that people are more likely to display preventive behaviors than in the first wave when COVID-19 arrived in Japan and did not spread significantly. During the sixth wave (Figure 1), the level of preventive behaviors temporarily returned to the levels observed during the early stages of the first wave when the first declaration of a state of emergency had been terminated. A significantly positive sign of *EMERGENCY* is observed in columns (2) and (3), which is reasonable because people were strongly urged not to go out. 

Table 7 presents different specifications where the second shot dummy is used to examine the effect of the second shot instead of using four dummies to capture the timing of the second shot. Table 7 only focuses on whether respondents received their second shot. We report the key variables, although the set of control variables is the same as that in Table 6, and the results are similar to that of Table 6. Columns (1)–(3) show significant positive signs for *VACCINE SECOND*, but columns (4) and (5) do not. Its absolute values of coefficient and statistical significance are larger for *VACCINE SECOND* than for *VACCINE FIRST.*

**Table 7:** FE model. Dependent variables are preventive behaviors.

	(1) *STAYING INDOORS*	(2) *NOT GOING TO WORK*	(3) *NOT FOR LEISURE*	(4) *HANDWASHING*	(5) *WEARING MASK*
*VACCINE FIRST*	0.057 ** (0.007–1.077)	0.032 (−0.015–0.080)	0.028 (−0.014–0.069)	0.028 ** (0.0003–0.057)	0.003 (−0.044–0.050)
*VACCINE SECOND*	0.107 *** (0.059–0.154)	0.090 *** (0.028–0.152)	0.079 *** (0.0355–0.123)	0.008 (−0.024–0.041)	0.005 (−0.038–0.048)
Adj R^2^ Obs.	0.52 54,007	0.66 54,007	0.37 54,007	0.62 54,007	0.49 54,007

Note: Numbers within parentheses are 95% CI. The set of control variables used in Table 6 is included, although the results are not reported. *** *p* < 0.01, ** *p* < 0.05.

Table 8 shows the results of the FE-ordered logit model. To interpret the results correctly, one needs to consider the marginal effects on the probability that respondents select a particular option [33,34]. For instance, they choose “1” for the question about the degree of “staying indoors” if respondents have not completed staying indoors at all. We can calculate how the first vaccination is correlated with this probability. The marginal effect of *VACCINE_SECOND* for (Prob[y = 1]) is −0.036 and is statistically significant at the 1 % level. This implies that the second vaccination reduced the probability that they had not completed staying indoors at all by 3.6%. Similarly, they choose “5” for the question on the degree of “staying indoors” if respondents have completely achieved staying indoors. Column (1) of Table 8 shows that *VACCINE_SECOND* for (Prob[y = 5]) is 0.021 and is statistically significant at the 1% level. This implies that the second vaccination increased the probability that they had completely achieved by staying indoors by 2.1%. The probability of choosing 2, 3, and 4 is also presented. Overall, *VACCINE_SECOND* shows statistical significance at the 1% level, with the exception of column (4). Further, the sign of *VACCINE_SECOND* is positive for Prob[y = 5] and negative for Prob[y = 1, 2]. Concerning (Prob[y = 3, 4]), its sign varies according to the columns. In contrast, *VACCINE_FIRST* shows similar results in columns (1), (4), and (5). However, it is not statistically significant at the 1% level, and the absolute values of the marginal effect are smaller than *those of VACCINE_SECOND.* Overall, the implications from the results of Table 8 are almost the same as those of Table 7. The results of the simple FE model can be more convenient and more intuitively interpreted than the FE-ordered logit model. Therefore, a simple FE model was used for the estimation in Table 9 and Table 10.

**Table 8:** FE Ordered logit model. Dependent variables are preventive behaviors.

	(1) *STAYING INDOORS*	(2) *NOT GOING TO WORK*	(3) *NOT FOR LEISURE*	(4) *HANDWASHING*	(5) *WEARING MASK*
*VACCINE FIRST*					
(Prob[y = 1])	−0.019 ** (−0.036–−0.003)	−0.016 (−0.047–0.014)	−0.003 (−0.011–0.004)	−0.003 ** (−0.006–−0.001)	−0.009 * (−0.019–0.0004)
(Prob[y = 2)	−0.010 ** (−0.019–−0.001)	−0.017 (−0.004–−0.001)	−0.002 (−0.006–0.002)	−0.007 ** (−0.013–−0.001)	−0.006 * (−0.014–0.0003)
(Prob[y = 3)	−0.0007 ** (−0.001–−0.0001)	0.0004 (−0.0003–0.001)	−0.007 (−0.024–0.009)	−0.022 ** (−0.041–−0.003)	−0.014 * (−0.029–0.0006)
(Prob[y = 4)	0.019 ** (0.003–0.036)	0.002 (−0.001–0.005)	−0.002 (−0.005–0.002)	−0.006 ** (−0.012–−0.001)	−0.015 * (−0.032–0.007)
(Prob[y = 5)	0.011 ** (0.001–0.021)	0.011 (−0.013–0.046)	0.015 (−0.018–0.049)	0.004 ** (0.007–0.073)	0.047 * (−0.002–0.096)
*VACCINE SECOND*					
(Prob[y = 1])	−0.036 *** (−0.054–−0.018)	−0.050 *** (−0.084–−0.015)	−0.012 *** (−0.020–−0.004)	−0.002 (−0.005–0.001)	−0.014 *** (−0.023–−0.004)
(Prob[y = 2)	−0.019 *** (−0.029–−0.009)	−0.005 *** (−0.008–−0.001)	−0.007 *** (−0.011–−0.002)	−0.004 (−0.010–0.001)	−0.010 *** (−0.017–−0.003)
(Prob[y = 3)	−0.001 *** (−0.002–−0.0006)	0.001 *** (0.0003–0.002)	−0.027 *** (−0.043–−0.010)	−0.013 (−0.032–0.005)	−0.021 *** (−0.035–−0.006)
(Prob[y = 4)	0.036 *** (0.018–0.053)	0.005 *** (0.001–0.009)	−0.006 *** (−0.010–0.002)	−0.004 (−0.009–0.002)	−0.023 *** (−0.039–0.006)
(Prob[y = 5)	0.021 *** (0.010–0.031)	0.048 *** (0.015–0.081)	0.053 *** (0.020–0.086)	0.023 (−0.010–0.057)	0.068 *** (0.020–0.115)
Wald-chi ^2^ Obs.	2551 54,007	1269 54,007	2340 54,007	1232 54,007	3426 54,007

Note: Numbers within parentheses are 95% CI. Values without parentheses are marginal effects. The set of control variables used in Table 6 is included, although the results are not reported. *** *p* < 0.01, ** *p* < 0.05, * *p* < 0.10.

### 3.2. Subsample Estimations (Young vs. Old Ages Groups)

A previous study of preventive behaviors in Japan divided the sample into <40 years and ≥40 years and found a difference between the samples [53]. We used the threshold to divide the samples because consumption inequality starts to increase at the age of 40 [54], which might influence individuals’ behaviors. Therefore, Table 5 and Table 6 report the results based on subsamples below 40 and subsamples equal to or over 40. Here, we focus on the key variables, although the same set of control variables is included. 

Contrary to the results in Table 6, Table 9 indicates the negative sign of vaccination dummies for staying indoors and not going out for work. Particularly, all dummies for the second shot are statistically significant for the estimations of not going out for work. Furthermore, the absolute values of the coefficients for *VACCINE SECOND_1, VACCINE SECOND_2, VACCINE SECOND_3,* and *VACCINE SECOND_4* were 0.249, 0.392, 0.347, and 0.615, respectively, suggesting that the vaccinated people are more likely to go to work or school than the unvaccinated ones as time passes. Moreover, these values are remarkably larger than those for staying indoors. Meanwhile, considering the results for not participating in leisure activities outside the home as a dependent variable, we did not observe statistical significance in the vaccination dummies.

**Table 9:** FE model: Dependent variables are preventive behaviors (ages ≤ 40 years).

	(1) *STAYING INDOORS*	(2) *NOT GOING TO WORK*	(3) *NOT FOR LEISURE*	(4) *HANDWASHING*	(5) *WEARING MASK*
*VACCINE FIRST*	−0.095 (−0.127–0.108)	−0.037 (−0.167–0.092)	−0.025 (−0.143–0.093)	−0.031 (−0.056–0.092)	0.017 (−0.053–0.027)
*VACCINE SECOND_1*	−0.106 (−0274–0.60)	−0.249 *** (−0.386–−0.111)	0.049 (−0.083–0.187)	−0.060 (−0.068–0.111)	0.021 (−0.059–0.025)
*VACCINE SECOND_2*	−0.283 ** (−0.556–−0.010)	−0.392 ** (−0.594–−0.191)	0.029 (−0.210–0.269)	0.0002 (−0.069–0.176)	0.053 (−0.074–0.031)
*VACCINE SECOND_3*	−0.288 * (−0.600–0.022)	−0.347 * (−0.713–0.017)	−0.079 (−0.337–0.179)	−0.053 (−0.129–0.413)	0.142 (−0.101–0.013)
*VACCINE SECOND_4*	−0.467 (−1.086–0.151)	−0.615 *** (−0.875–−0.355)	−0.140 (−0.740–0.458)	0.004 (−0.116–0.245)	0.064 (−0.243–0.088)
Adj R^2^ Obs.	0.49 15,407	0.56 15,407	0.37 15,407	0.52 15,407	0.58 15,407

Note: Numbers within parentheses are 95% CI. The set of control variables used in Table 6 is included, although the results are not reported. *** *p* < 0.01, ** *p* < 0.05, * *p* < 0.10.

Table 10 shows the results of the older generation show a similar of dummies for vaccination are very similar to those in Table 6. As a whole, the estimation results are robust to alternative specifications. 

**Table 10:** FE model: dependent variables are preventive behaviors (ages > 40 years).

	(1) *STAYING INDOORS*	(2) *NOT GOING TO WORK*	(3) *NOT FOR LEISURE*	(4) *HANDWASHING*	(5) *WEARING MASK*
*VACCINE FIRST*	0.036 (−0.022–0.095)	0.026 (−0.034–0.086)	0.013 (−0.030–0.056)	0.024 * (−0.003–0.051)	−0.012 (−0.003–0.051)
*VACCINE SECOND_1*	0.089 *** (0.036–0.143)	0.105 ** (0.040–0.170)	0.051 * (−0.001–0.103)	−0.001 (−0.046–0.043)	−0.016 (−0.046–0.043)
*VACCINE SECOND_2*	0.120 *** (0.053–0.187)	0.175 *** (0.097–0.252)	0.084 *** (0.023–0.144)	−0.024 (−0.074–0.025)	−0.021 (−0.074–0.025)
*VACCINE SECOND_3*	0.118 * (− 0.002–0.239)	0.170 *** (0.058–0.282)	0.005 (− 0.123–0.135)	0.012 (−0.052–0.078)	−0.043 (−0.052–0.078)
*VACCINE SECOND_4*	0.131 (−0.219–0.481)	0.167 * (−0.024–0.358)	−0.123 (−0.384–0.136)	−0.048 (−0.211–0.115)	−0.077 (−0.211–0.115)
Adj R^2^ Obs.	0.52 38,600	0.68 38,600	0.35 38,600	0.63 38,600	0.53 38,600

Note: Numbers within parentheses are 95% CI. The set of control variables used in Table 6 is included here, but the results are not reported. *** *p* < 0.01, ** *p* < 0.05, * *p* < 0.10.

## 4. Discussion

Using independently collected panel data, we found that vaccinated individuals are more likely to stay at home, frequently wash their hands, and wear masks than unvaccinated individuals, consistently from the early stages of COVID-19 to after vaccine distribution. The results obtained by analyzing the FE model indicate that the gap between vaccinated and unvaccinated individuals in terms of “staying at home” increased. In the context of “handwashing” or “wearing a mask,” the gap did not reduce. 

Considering the results using both subsamples, vaccinated people in the young group are more motivated to go to work or school than those who are not vaccinated; however, they do not have a stronger motivation to go out for leisure. We found that they received the shot in the workplace or school and were encouraged or required to go out for work or education. The incentive that young people have is not common for aged groups because they consist mainly of retired older people. The sample size and observations of vaccinated people in the old group were far larger than those for the young group. Hence, the influence of vaccination dummies for the old group outweighs that of the young group (Table 6).

Previous related studies were conducted in various countries. Vaccination reduces the risk of contracting COVID-19, which might lessen preventive behaviors. Consistent with the conjecture, vaccinated people became less likely to engage in preventive behaviors in China [25] and Bangladesh [26]. As opposed to it, individuals’ COVID-19 preventive behaviors did not decrease after vaccination in the UK [28,55], China [27]. These findings are consistent with those of the present study. This indicates that preventive behaviors against COVID-19 are motivated by self-interest and other related concerns. This is in line with the expectation that the COVID-19 vaccine protects vaccinated individuals and society by reducing disease transmission. 

Public health messages from governments/scientists are considered to encourage vaccinated people to continue to engage in preventive behaviors. In other words, information was diffused through various media, calling for a cautious attitude, which possibly formed social norms to engage in preventive behaviors. People display preventive behaviors that depend on caring and fairness concerns [56,57]. Vaccination reduced people’s frequency of hand washing and their intensity of physical distancing but did not influence the rate of mask-wearing [58]. In our interpretation, mask-wearing can be more easily monitored by others, which might cause people to wear masks. Vaccinated people will be criticized by members of society if they do not engage in preventive behaviors. Peer pressure is enforced by the mechanism that vaccinated individuals who engage in socially anticipated behaviors exhibit less generosity toward unvaccinated individuals [59]. This increased the incentive of vaccinated individuals to engage in preventive behaviors but decreased their subjective well-being. We encountered the difficult problem of balancing social and individual’s interests. 

A closer examination found that young individuals aged ≥ 40 years tended to go out to work post-vaccination. They are likely to be vaccinated at their workplace so they can work safely. They need to go to work as working from home is yet to be firmly established in Japan, and they are also less likely to hold a management position, which makes working from home possible. Inevitably, vaccinated young workers seem reluctant to obey the norms. However, apart from going out to work, they continued displaying other preventive behaviors, such as refraining from participating in leisure activities outside the home, frequently washing their hands, and wearing masks. In China, older people were less likely to stay at home during the peak of the pandemic because they could not sufficiently acquire food and basic services online [60]. As opposed to this, we found that older people were more likely to stay at home in Japan. This might be because older people are more able to use the internet and more willing to reduce the risk of being infected while outside in Japan than in China. 

### 4.1. Strength

Many research papers have investigated preventive behaviors against COVID-19. However, most of the studies are based on cross-section data rather than panel data [61,62]. Hence, these studies suffered omitted variable biases and did not explore causality between dependent variables and preventive behaviors. According to Liang et al. [63], even after vaccination, preventive behaviors against COVID-19 are important because there is no guarantee of full protection from COVID-19 [61,64]. However, it is not known whether preventive behaviors depend on completing vaccination [61,62,63]. This study’s contribution is to explore the correlation between vaccination and preventive behaviors by comparing before and after vaccination using individual-level panel data.

Overall, our key findings are consistent with the argument that “individuals act upon the social contract. The stronger they perceive it as a moral obligation, the more they act upon it. Emphasizing the social contract could help increase vaccine uptake, prevent free riding, and eventually support the elimination of infectious diseases.” [59].

Our findings provide the following policy recommendations.: first, the government and scientists should send messages to strengthen social norms to maintain caution against COVID-19 before herd immunity is realized. Further, rapidly increasing the rate of vaccinations within 2 months after the commencement of vaccination diffusion is critical for developing herd immunity as vaccinated people become less likely to stay at home after 3 months have passed. The sample used in this study is representative of the Japanese population. Therefore, the conclusions can be extrapolated to Japan as a whole.

### 4.2. Limitation

The way to divide the sample according to age depends on the aim of the study. Those who are over 60 ages may show great differences in preventive behaviors from those who are under the age of 60 because of work status, income level, and other reasons. In this study, 40-year-olds are selected as the basis for age stratification. In Japan, consumption inequality within a fixed cohort grows with age. Particularly consumption inequality starts to increase at the age of 40 [54]. Hence, we placed focus on the difference between the middle or older generations and younger generations. However, it is valuable to compare results between retired generations and working generations. 

As time goes by, the prevention behavior will also change. Unfortunately, due to the data covering this limited period, we cannot scrutinize how the behavior changes, such as staying at home, wearing mask, and washing hands, were affected by vaccination from a long-term perspective. To this end, it is necessary to construct the data covering longer periods by pursuing identical participants.

INTAGE gave incentives to the participants. However, there is a possibility that we collected inaccurate information. Fake participants possibly were included because payment from INTAGE gave them an incentive. It is critical to improve transparency to solve ethical problems, and empirical researchers should develop a system to avoid these problems.

Selection bias may have occurred because those who pay more attention to COVID-19 are more likely to participate in the surveys. People may be motivated to follow socially desired responses when answering questions, resulting in biases. Despite using panel data, causality between vaccination and preventive behaviors has not been scrutinized. In order to examine the causality, an experimental study to control endogenous biases should be called for. Further, careful attention should be given to the fact that those without internet access were underrepresented. These are the limitations of the present study.

## Figures and Tables

**Table 1:** Definitions of key variables.

Variables	Definition
	Dependent variables
*STAYING I* *NDOORS*	In the last week, how consistent were you at “not going out of home?” Please choose among five choices. 1 (not completed at all) to 5 (completely consistent).
*NOT GOING TO WORK*	In the last week, how consistent were you at “not going out to work (or school)?” Please choose among five choices. 1 (not completed at all) to 5 (completely achieved).
*NOT FOR LEISURE*	In the last week, how consistent were you at “not going out to events or travel?” Please choose among five choices. 1 (not completed at all) to 5 (completely achieved).
*HANDWASHING*	In the last week, how consistent were you at “washing your hands?” Please choose among five choices. 1 (not completed at all) to 5 (completely achieved).
*WEARING MASK*	In the last week, how consistent were you at “wearing a mask?” Please choose among five choices. 1 (not completed at all) to 5 (completely achieved).
	Confounders (Independent variables)
*VACCINE FIRST*	Did you get the first shot (but not the second one)? 1 (Yes) or 0 (No)
*VACCINE SECOND*	Did you get the second shot? 1 (Yes) or 0 (No)
*VACCINE SECOND_1*	Did you get the second shot this month? 1 (Yes) or 0 (No)
*VACCINE SECOND_2*	Did you get the second shot last month? 1 (Yes) or 0 (No)
*VACCINE SECOND_3*	Did you get the second shot two months ago? 1 (Yes) or 0 (No)
*VACCINE SECOND_4*	Did you get the second shot three months ago? 1 (Yes) or 0 (No)
*PROB_COVID19*	What percentage do you think is the probability of your getting COVID-19? Please choose from 0 to 100 (%)
*SEVERITY COVID19*	How serious are your symptoms if you are infected with the novel coronavirus? Choose from 6 choices. 1 (very small influence) to 6 (death)
*EMERGENCY*	Is your area in a state of emergency? 1 (Yes) or 0 (No)
*FEAR*	How intense is your feeling of fear? Please answer on a scale from 1 (I have not felt this emotion at all) to 5 (I have felt this emotion strongly).
*ANXIETY*	How intense is your feeling of anxiety? Please answer on a scale from 1 (I have not felt this emotion at all) to 5 (I have felt this emotion strongly).
*ANGER*	How intense is your feeling of anger? Please answer on a scale from 1 (I have not felt this emotion at all) to 5 (I have felt this emotion strongly).
*AGE*	Ages
*MALE*	Select 1 if you are male and 0 if otherwise.
*UNIVERSITY*	Select 1 if you graduated from university and 0 if otherwise.

**Table 2:** Sample size (observations) and loss rates for each survey.

Waves	Obs.	Loss Rate %
1	4359	0
2	3495	19.8
3	4013	7.9
4	3996	8.3
5	3877	11.1
6	3626	16.8
7	3491	19.9
8	3509	19.5
9	3529	19.0
10	3440	21.1
11	3304	24.2
12	3280	24.8
13	3392	22.2
14	3349	23.2
15	3347	23.2

## Data Availability

The datasets used and analyzed in this study are available from the corresponding author upon reasonable request.

## Supplementary Materials

The following supporting information can be downloaded at: https://www.mdpi.com/article/10.3390/vaccines11040810/s1. Supplementary File S1, including Figure S1 and Figure estimation results of balanced panel data, was exhibited in Supplementary File S2. We provided the original questionnaire (in Japanese) in Supplementary File S3_JP. In addition, the English version is also added as S3_EN.
