# Supplementary material for: Association between the COVID-19 Vaccine and Preventive Behaviors: Panel Data Analysis from Japan
Source: Vaccines (Basel). 2023 Apr 6;11(4):810. doi: 10.3390/vaccines11040810 (PMC10145704; doi:10.3390/vaccines11040810)
Supplement: Supplementary file 1 [file vaccines-11-00810-s001.zip › Supplementary S1.pdf]

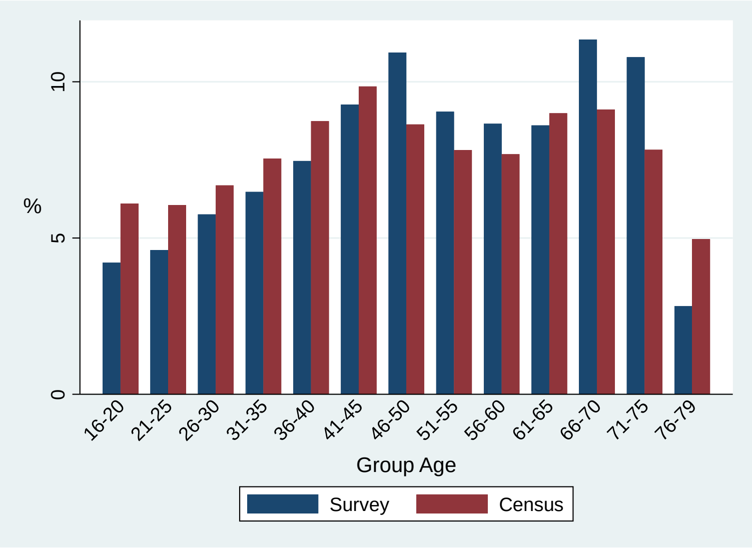

Figure S1. Comparison of the age distribution of our survey sample with that of the 2015 Japan population census.

Sources: Statistics Bureau, Ministry of Internal Affairs and Communications (2015). 2015 Japan Population Census.

https://www.e-stat.go.jp/stat-search/files?page=1&layout=datalist&toukei=00200521&tstat=000001080615&cycle=0&tclass1=000001089055&tclass2=000001089056&result_page=1&second=1&second2=1 (accessed on February 18, 2018).

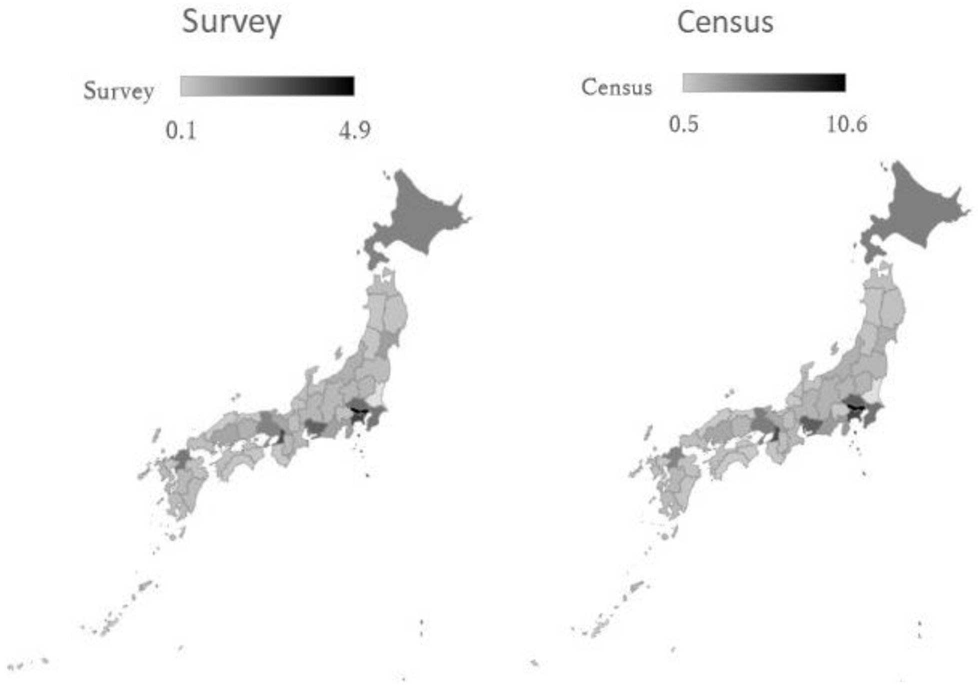

Figure S2. Comparison of the geographical distribution of our survey sample with that of the 2015 Japan population census.

Note: Darker shaded areas indicate more populated areas. Number in the map indicates the percentage of population in each residential prefecture.

Sources: Statistics Bureau, Ministry of Internal Affairs and Communications (2015). 2015 Japan Population Census.

https://www.stat.go.jp/data/nihon/02.html

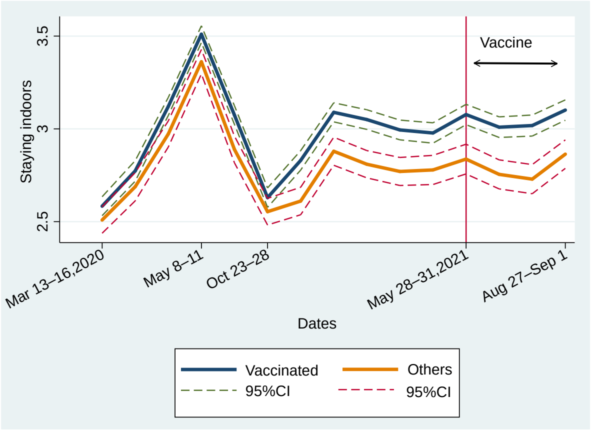

Figure S3(a). Change in staying indoors.

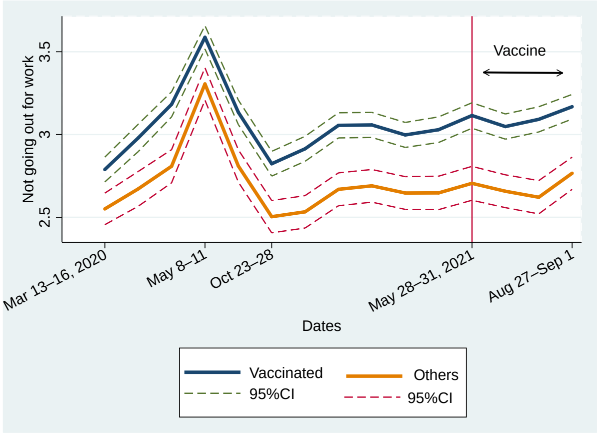

Figure S3(b). Change in not going out for work.

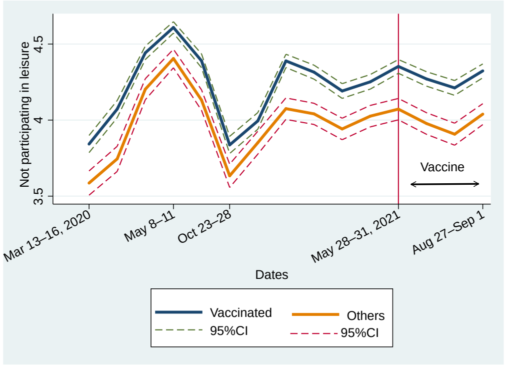

Figure S3(c). Change in not participating in leisure activities outside home.

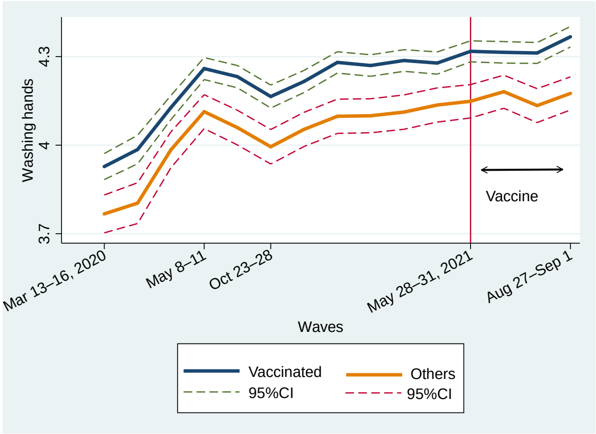

Figure S3(d). Change in handwashing.

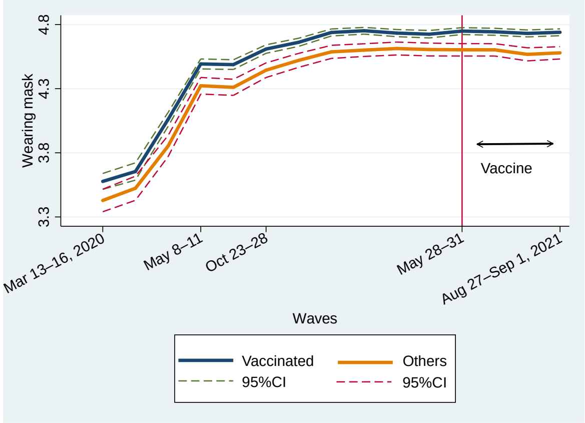

Figure S3(e). Change in wearing mask.
