# Supplementary material for: Association between the COVID-19 Vaccine and Preventive Behaviors: Panel Data Analysis from Japan
Source: Vaccines (Basel). 2023 Apr 6;11(4):810. doi: 10.3390/vaccines11040810 (PMC10145704; doi:10.3390/vaccines11040810)
Supplement: Supplementary file 1 [file vaccines-11-00810-s001.zip › Supplementary S2.pdf]

Table S1. FE model. Dependent variables are preventive behaviors. Same specification of Table 6. Sample including those who stopped participating in the surveys during the studied period.

	(1) *STAYING INDOORS*	(2) *NOT GOING TO WORK*	(3) *NOT FOR LEISURE*	(4) *HANDWASHING*	(5) *WEARING MASK*
VACCINE FIRST	0.046	0.012	0.018	0.005	0.014
VACCINE SECOND_1	0.085**	0.064**	0.083**	−0.006	−0.010
VACCINE SECOND_2	0.125**	0.098***	0.111**	0.002	0.002
VACCINE SECOND_3	0.102	0.043	0.115	−0.014	−0.014
VACCINE SECOND_4	0.020	0.024	0.063	−0.038	−0.038
Adj R^2	0.55	0.42	0.69	0.53	0.65
Obs.	30,120	30,120	30,120	30,120	30,120

Note: CI is redundant information and so not reported. For convenience, the coefficient of PROBABILITY COVID-19 is multiplied by 1000. Although the effects are not reported, the model includes various control variables used in the specification of Table 6, and CI are calculated.

***p<0.01

**p<0.05

*p<0.10
