# Supplementary material for: Association between the COVID-19 Vaccine and Preventive Behaviors: Panel Data Analysis from Japan
Source: Vaccines (Basel). 2023 Apr 6;11(4):810. doi: 10.3390/vaccines11040810 (PMC10145704; doi:10.3390/vaccines11040810)
Supplement: Supplementary file 1 [file vaccines-11-00810-s001.zip › Supplementary S3_EN.pdf]

Questionnaire on the Novel Coronavirus

This survey aims to clarify how you perceive the threat of the novel coronavirus and prevent it, and use the obtained knowledge to cope with the occurrence of similar event in the future. To this aim, we also ask your attributes, preferences, and opinions in the questionnaire. Your responses will be used only for academic purpose. Thank you very much for your cooperation.

QA1. To what degree are you currently feeling happiness? On a scale of 0-10 where “10” is “very happy” and “0” is “very unhappy,” how do you rate your current level of happiness? **(X ONE Box)**

Very Unhappy ←——————→ Very Happy

0☐ 1☐ 2☐ 4☐ 5☐ 6☐ 7☐ 8☐ 9☐ 10☐

QA2. In the last two weeks, how much of the following items have you achieved? Please indicate from 1(I have not achieved at all) to 5 (I have completely achieved).

1. Not going out of home ……………………..…………….…………….. 1 2 3 4 5
2. Not going to a working place or school ………………………..…….. 1 2 3 4 5
3. Wearing a mask …………………………………..……..……….. 1 2 3 4 5
4. Washing hands with great care …………………………..………….. 1 2 3 4 5
5. Cancelling scheduled trips and stopping participation in an event ….. 1 2 3 4 5

QA3. In the last two weeks, how much have you felt the emotion of anger, fear, and anxiety? Please indicate from 1 (I have not felt the emotion the slightest bit during the last week) to 5 (I have felt the emotion more strongly than ever during the last week).

Anger ……………………………………..………….. 1 2 3 4 5
Fear ………………………………………..………. ... 1 2 3 4 5
Anxiety ……………………………………………….. 1 2 3 4 5

QA4. In the last two weeks, have any infected people been detected in the places specified below?

1 Your neighbor …………………………. Yes No don’t know
2 Your relatives and friends ……………. Yes No don’t know
3 Your working place and school ………. Yes No don’t know
4 The Municipality where you live …………. Yes No don’t know
5 The Prefecture where you live ……………. Yes No don’t know

QA5. **Assume that** a vaccine, which is verified to be effective against novel coronavirus is available at the cost of 100 thousand yen (the cost is not covered by insurance)? Would you take it after paying 100 thousand yen? If you definitely do not take the vaccine, you would choose "1", and if you definitely take, you would choose "5" in the scale of 1 to 5. **(X ONE Box)**

Definitely do not take ←→ **Definitely take**

1 2 3 4 5

QA6. What do you expect is the probability (%) of you getting infected with the novel coronavirus within one month?

. %

QA7. How serious are your symptoms if you are infected with the novel coronavirus? **(X ONE Box)**

1 Symptoms that have little influence

2 A symptom that takes you about a few days to recover from

3 A symptom that takes you about a week to recover from

4 A serious symptom that could take about a month to recover from

5 A very serious symptom that could cause some after-effects

6 An extremely serious symptom that could cause death

QA8. What do you expect from the spread of infection of the novel coronavirus in Israel within one month? Please choose one of the following options and check one box.

1 The infection is completely converged.

2 The infection is heading toward convergence.

3 The infection does not spread more than it already has.

4 Though the number of deaths and infected persons increase, serious situations don't occur.

5 Though the infection spreads all over and deaths accumulate, paralysis of urban functions and the economy is limited.

6 The infection spreads all over Israel and deaths accumulate, completely paralyzing urban functions and the economy.

QA9. Do you think that the following measures taken to cope with the novel coronavirus infection in these two weeks were appropriate? Please indicate for every item from 1=too lax (or too late), strong opposition, 2=lax, slight opposition, 3=very appropriate and decisive judgement, 4= strict (or hasty, slight opposition), 5=went too far (or too hasty, strong opposition)

Please change the items to meet the situation in Israel!

1. Admission of overseas travelers of entrance from abroad
2. Government's request for closure of public schools.
3. Cancelling admission to Sports games
4. Staggered working hours and teleworks
5. Inspection and medical system

QA10. What do you expect from your revenue and GDP in Israel and the world, in the coming two years (2020 and 2021) in comparison with 2019? Please choose from
1=improve by more than 4%, 2=improve between 1% to 3.99%, 3= almost unchange, 4=drop between 1 to 3.99%, 5=drop between 4 to 9.99%, 6= drop more than 10%

	Coming one year (2020)	the next year (2021)
Revenue of your household		
GDP of Israel		
GDP of the world		

QA11. In these two weeks, have you received enough information about the Coronavirus? Please choose from 1 (definitely not enough) to 5 (definitely enough).

QA12. What do you expect the probability (%) is of cancelling the Tokyo Olympic Games in July and August 2020?

__________%

QB1. Your gender:

1 Male 2 Female

QB2. Your age:

years old

Tens ones

QB3. Please indicate your relationship status by selecting the appropriate option.

1 Married (including common-law marriage)
2 Divorced
3 Widowed
4 Unmarried
5 Cohabitation

QB4. Please indicate the highest level of education completed by you and your parents. *If you are still in school, please select the number that corresponds to the degree you are pursuing right now.* **(X ONE Box for each column)**

You **your father** **your mother**

1. Grade School
2. High School
3. College – Associate's Degree (2 years)
4. College – Bachelor's Degree (4 years)
5. Masters Degree
6. Doctoral Degree

QB5. What is your current occupation? **(X ONE Box)**

1 Full-time worker
2 Part-time worker
3 Housewife/Househusband(unemployed)
4 Student
5 Retired (excluding housewives/househusbands)
6 Unemployed (excluding housewives/househusbands)
7 Other

Please replace with the Israeli style question and using ILS

QB6. Approximately state the earned annual income (before taxes, with bonuses) of your entire household for 2019. *(If you are a student, please select the income of your parents' entire household.)* **(X ONE Box)**

01 ☐ Less than $10,000 07 ☐ $100,000 to less than $120,000

02 ☐ $10,000 to less than $20,000 08 ☐ $120,000 to less than $140,000

03 ☐ $20,000 to less than $40,000

04 ☐ $40,000 to less than $60,000

05 ☐ $60,000 to less than $80,000

06 ☐ $80,000 to less than $100,000

09 ☐ $140,000 to less than $160,000

10 ☐ $160,000 to less than $180,000

11 ☐ $180,000 to less than $200,000

12 ☐ More than $200,000

13 ☐ don' t want to answer

QB7. How many people are currently living in your household?

# of people: _____ _____

Tens ones

QB8. Are the following people included in your family? If yes, please check the Box.

☐1. Children going to elementary school

☐2. Children going to junior high school

☐3. Grand children going to elementary school

☐4. Grand children going to junior high school

☐5. Elderly people between 70 and 80 years

☐6. Elderly people over 80 years

QB9. In which region (state) do you live now?

Name of the region (state) in Israel: ________________________________

QB10. Do the following statements hold true for you? Please choose from 1 (it is particularly true for you) to 5 (it doesn't hold true at all for you). Of course, you may choose any number in between as well. **(X ONE Box For EACH)**

1. I have anxiety about my health …………………….. 1 2 3 4 5
2. I am deeply religious……………………………………1 2 3 4 5
3. I have undergone a periodic blood test ………………1 2 3 4 5
4. In general, most people are trustworthy …………….. 1 2 3 4 5
5. In uncertain times, I usually expect the best ………….. 1 2 3 4 5
6. If something can to go wrong for me, it will ……………. 1 2 3 4 5
7. I am always optimistic about my future. …………………1 2 3 4 5

QB11. Please indicate if you are affiliated with any of the following religions. **(X ONE Box)**

01 □ None

02 □ Judaism

03 □ Islam

04 □ Christian

05 □ Buddhism

06 □ Others not listed above

07 □ Prefer not to answer

QB12. On a scale of 0-10, “10” being the “Wealthiest” and “0” being the “Poorest”, please indicate what you think your standard of living is and what you think the standard of living was for your family when you were 15 years old. If you prefer not to answer, please check this Box. **(X ONE Box)**

Poorest ↔ **Wealthiest**

Now 0 □ 1 □ 2 □ 4 □ 5 □ 6 □ 7□ 8 □ 9 □ 10□ prefer not to answer □

15 years old 0 □ 1 □ 2 □ 4 □ 5 □ 6 □ 7□ 8 □ 9 □ 10□ prefer not to answer □

Please change into NIS

QB13. Assume that you know there is a 50% chance of losing $1000 on a given day. You can take out insurance to cover this amount in case of a loss. What is the most you would pay to purchase the insurance? (X ONE Box)

1 Do not purchase even if the prices is $0

2 Purchase if the price is less than or equal to $10

3 Purchase if the price is less than or equal to $50

4 Purchase if the price is less than or equal to $100

5 Purchase if the price is less than or equal to $150

6 Purchase if the price is less than or equal to $200

7 Purchase if the price is less than or equal to $300

8 Purchase if the price is less than or equal to $400

9 Purchase if the price is less than or equal to $500

10 Purchase even if the price is more than $500

Please change into NIS

QB14. Assume that you know there is a 0.1% chance of losing $0.5 million on a given day. You can take out insurance to cover this amount in case of a loss. What is the most you would pay to purchase the insurance? (X ONE Box)

1 Do not purchase even if the prices is $0
2 Purchase if the price is less than or equal to $10
3 Purchase if the price is less than or equal to $50
4 Purchase if the price is less than or equal to $100
5 Purchase if the price is less than or equal to $200
6 Purchase if the price is less than or equal to $300
7 Purchase if the price is less than or equal to $500
8 Purchase if the price is less than or equal to $1000
9 Purchase if the price is less than or equal to $5000
10 Purchase if the price is less than or equal to $10000
11 Purchase even if the price is more than $10000

QB15. Now let's assume that you have the option to receiveNIS300 now or receive a different amount in twelve months. *Compare the amounts and timing in Option "A" with Option "B" and indicate which amount you would prefer to receive for all 8 choices*

**Option "A"** Receive now	**Option "B"** Receive in 12 months	*An Annual Interest Rate of*	Which ONE do you prefer? (X ONE Box For EACH Row)	
300	285	−5%	A	B
300	300	0%	A	B
300	306	2%	A	B
300	312	4%	A	B
300	318	6%	A	B
300	330	10%	A	B
300	360	20%	A	B
300	420	40%	A	B

QB16. Assuming you find a well-known charity that provided financial help to people who typically had about one-fifth of your family income per person. Up to how much of your own family income per month, would you be willing to give the charity, if you knew the charity would give twice the amount of your donation directly to benefit these people? (**X ONE Box)**

1 No help at all
2 Up to 2 % of your family income per month
3 Up to 5 % of your family income per month
4 Up to 10 % of your family income per month
5 Up to 20% of your family income per month
6 More than 20% of your family income per month

QB17. Usually, what is the common source of news for you. Please indicate the item most frequently used with 1, and the next item with 2.

□ newspaper □ news program of TV

□ conversation with family and friends face to face or communications

SNS (social networking service; including twitter)

news in web □ news through applications in smartphone

QB18 In terms of taking risks in your day to day life, how would you classify yourself on a scale of 1–10? Please note that 1 indicates that you avoid taking any risks at all costs and 10 indicates that you engage in extremely risky and dangerous behaviors.

avoid taking any risks at all costs ← → **engage in extremely risky and dangerous behaviors**

1 □ 2 □ 4 □ 5 □ 6 □ 7□ 8 □ 9 □ 10□
