# Supplementary material for: Association between the COVID-19 Vaccine and Preventive Behaviors: Panel Data Analysis from Japan
Source: Vaccines (Basel). 2023 Apr 6;11(4):810. doi: 10.3390/vaccines11040810 (PMC10145704; doi:10.3390/vaccines11040810)
Supplement: Supplementary file 1 [file vaccines-11-00810-s001.zip › Supplementary S3_JP.pdf]

新型コロナウイルスに関するアンケート調査

本調査では、日本在住の皆様が新型コロナ感染の問題をどのように考え、対処しているかをお尋ねして、その実態を把握することを目的としています。具体的に、皆様の日々のくらしの好みや考え方をお尋ねする質問と共に、健康状態や新型コロナウイルス感染症の感染予防に関する考え方をお尋ねする質問が含まれます。本調査でご回答いただいた内容は全て統計的に処理され、個人が特定できない形式に変換して取り扱われます。回答の要約データや原データが公開される可能性はありますが、その場合にも個人を特定できる情報と結びつけて公開されることは絶対にありません。また、データを本調査研究の目的以外に使用することは絶対にありません。それぞれの質問について、考えている通りにお答えください。
本調査への参加は強制ではありません。参加者の皆様は、調査の途中でも参加をやめることができます。本調査への回答を途中でやめたいと思った場合は、ブラウザを閉じてください。その際、謝礼ポイントは付与されませんので、ご了承頂けますようお願い申し上げます。本調査は、今後継続して5回実施される予定です。皆様には、5回全ての調査への継続参加を積極的に検討いただきますよう、お願い申し上げます。
なお、謝礼ポイントは、1回の調査の回答完了毎に付与されます。また、「第2回目」の実施は5月上旬を予定しております。
上記の事項をご確認頂き、本調査に参加くださる方は「次へ進む」をお選びください。
調査に参加されない方は、ここで終了となります（謝礼ポイントはつきません）。

A1　あなたは今どの程度幸福だと感じていますか。「非常に幸福」を 10 点、「非常に不幸」を０点として、あなたが今感じている幸福度は何点ぐらいになると思いますか。当てはまるものを１つ選び、番号に〇をつけてください。

非常に不幸　←――――――――――→　非常に幸福

0 □ 1 □ 2 □ 4 □ 5 □ 6 □ 7□ 8 □ 9 □ 10□

A2　この 2 週間、あなたは以下の事項をどれぐらい達成しましたか。 1 (=全く達成していない)から 5 (=完全に達成した)のうち当てはまるものを 1 つ選び 5 段階で答えてください。

1. 外出しない …………………………………………………... 1 2 3 4 5
2. 職場への出社・学校への登校をしない ……………...…… 1 2 3 4 5
3. マスクの着用 ………………………………………………… 1 2 3 4 5
4. 念入りな手洗いの励行 ……………………………………… 1 2 3 4 5
5. 計画していた旅行の中止、イベント参加の見合わせ ……. 1 2 3 4 5

D19　新型コロナ感染を防ぐために、あなたが自分に課していた行動制限は、ワクチンを打った後、きつくなりましたか、ゆるくなりましたか。以下の事項についてお答えください。
1(=非常にゆるくなった)　2(=ゆるくなった)　3(=変わらなかった) 4(=きつくなった) 5 (非常にきつくなった)

1. 外出しない程度 …………………………………………………... 1 2 3 4 5
2. 職場への出社・学校への登校をしない泥土 ……………...…… 1 2 3 4 5
3. マスクを着用する程度 ………………………………………………. 1 2 3 4 5
4. 念入りに手洗いをする程度 ……………………………………… 1 2 3 4 5
5. 計画していた旅行を中止したり、イベント参加を見合わせる程度…. 1 2 3 4 5

A3　この 2 週間、あなたは怒り、おそれ、不安、熱狂、期待感、といった感情をどれぐらい感じましたか? 1= (全く感じなかった) から 5 (=かつてないほど強く感じた) の 5 段階でお答えください。

怒り	1	2	3	4	5
恐れ	1	2	3	4	5
不安	1	2	3	4	5
熱狂	1	2	3	4	5
期待感	1	2	3	4	5

A5 日本でも始った新型コロナウイルスに対するワクチンについてお伺いします。このワクチンは無料で提供される予定ですが、あなたは、ワクチンが受けられることになったら接種しますか。絶対に接種しない場合を“1”、絶対に接種する場合を“5”として、5 段階でお答えください。
すでに接種した人は接種する前の気持ちを思い出してお答えくださるとともに、その回答に加えて、6 をチェックしてください。

1：絶対に接種しない← →5：絶対に接種する　6:すでに接種した

1　2　3　4　5

もし、このワクチンが 10 万円で提供されることになっていたら、あなたはどうしますか？

1：絶対に接種しない← →5：絶対に接種する　6:すでに接種した

1　2　3　4　5

もし､このワクチンを受けると 1 千円の補助金がもらえることになっていたらどうですか？

1：絶対に接種しない← →5：絶対に接種する　6:すでに接種した

1　2　3　4　5

もし、ワクチン接種をしないとレストラン、旅行、イベントに行くことなどが制限されるようになっていたらどうどうですか？

1：絶対に接種しない← →5：絶対に接種する　6:すでに接種した

1　2　3　4　5

もし､このワクチンを受けると 5 千円の補助金がもらえることになっていたらどうですか？

1：絶対に接種しない← →5：絶対に接種する　　6:すでに接種した

1　2　3　4　5

A15　日本においても接種が始まった新型コロナワクチンに関する各項目について、あなたはどのくらい重要だと思いますか？ 1 (=全く重要でない)、2(=あまり重要でない)、3(=どちらともいえない)、4(=かなり重要だ)、5 (=非常に重要だ)

1. 少しでも早期に接種が可能になること………………………….. 1　2　3　4　5

2. 接種費用が低額であること……………………………………….. 1　2　3　4　5

3. 副作用がないこと………………………………………………….. 1　2　3　4　5

4. 有効性が強いこと………………………………………………….. 1　2　3　4　5

5. どこでも簡便に接種できること………………………………….. 1　2　3　4　5

D17　新型コロナワクチンの接種についてどのようにお考えですか。あなたのお考えに最も近いもの 1 つを選んでお答えください。

1. すでに第 1 回目の接種を受けた。(受けたワクチン名＝ファイザー、モデルナ)

2. すでに 2 回の接種を受けた。

3. 接種の順番が来たら、ワクチンを受けるつもりである。

4. 接種を受けるかどうかまだ決めていない。

5. ワクチン接種を受けるつもりはない。

D18 新型コロナワクチンを接種したとき、あなたの副反応はどのようなものでしたか。あてはまるものすべてを選び、その状況を選らんでください。

1. 接種箇所がはれた　(1 回目、2 回目、両方)

2. 腕が上がりにくくなった　　(1 回目、2 回目、両方)

3. 37.5 度以上発熱した　(最高 37.5 度、38 度、38 度以上；1 日間, 2 日またはそれ以上)
(1 回目、2 回目、両方)

4. 安静にしていた（Y 日間）　(1 回目、2 回目)

5. 解熱剤を飲んだ　　(1 回目、2 回目、両方)

6. 医者の診察を受けた　　(1 回目、2 回目、両方)

C7. あなたがワクチンを受けるかどうかを決めるにあたって、下記の事柄はどのくらい重要ですか？　1 (=全く重要でない)、2(=あまり重要でない)、3(=どちらともいえない)、4(=かなり重要だ)、5 (=非常に重要だ) のうち、最もあなたの考えに近いものを 1 つお選びください。

1. 自分の命を守るため

2. 感染の連鎖を防ぐため.

3. 仕事や学校に安全に行けるようになるため

4. 自分から家族への感染リスクを小さくし、家族の命を守るため

5. レストランや旅行などに行けるようになるため

C6. 日本でも接種が始まった新型コロナウイルスのワクチンに関する以下の記述について、あなたは賛成ですか、反対ですか？1 （=完全に反対）、2(=反対)、3(=どちらでもない)、4(=賛成)、5(=完全に賛成)　から一つお選びください。

1.　 このワクチンは私の健康を害するように思われる。

2.　このワクチンは変異ウイルスには有効でない。

3.　このワクチンを受ける時間がない。

4.　どんなワクチンも嫌いだ。

5.　 もし、新型コロナにかかっても、開発中の新薬で回復するだろう

.

7.　 このワクチンの長期の副作用は有害かもしれない

8.　 このワクチンの接種について、どのよう優先順位もつけるべきではない: すべての人は平等に扱われなければならないから。

9.　　政府は他国に遅れることなく、ワクチンを供給するべきだ。

10.　政府は開発途上国の人々のワクチン接種に貢献すべきだ。

11.　ワクチン接種済証明カードを発行して、旅行や大規模イベントへの参加を認める要件として使うべきだ。

A6　この先 1 か月以内に、あなたが新型コロナウイルスに感染する確率はどれぐらいだと思いますか？もし、あなたが予防対策をとらなかった場合の確率と、もし、あなたが新型コロナウイルスのワクチンを接種できたとした場合の確率もお答えください。 また、周りの人が感染する確率はどれくらいだと思いますか？ ※回答は必須です。0〜100%の間でお答えください。　　　　　　　　　　　　　　　　　　　　%

あなたが予防対策をとらなかった場合の確率　　　　　　%

新型コロナウイルスのワクチンを接種できた場合の感染する確率

周りの人が感染する確率　　　　　　　　　　　　　　　　%

A7　あなたが新型コロナウイルスに感染した場合、どのくらいの病状になると思いますか。1つを選んでマークしてください。

1. たいした影響はない
2. 回復までに２～３日かかる
3. 回復までに１週間ほどかかる
4. 回復までに１ヶ月ほどかかる
5. 後遺症が残るほどに重篤になる
6. 死亡する可能性がある重篤な病気になる

A8　これから先の 1 か月間で、日本において、新型コロナウイルスの感染はどのぐらい広がると思いますか。以下から 1 つを選んでマークしてください。

1. 感染は完全に収束する
2. 感染は収束に向かう
3. 感染は現在の水準にとどまり、これ以上広がらない
4. 感染者数・死者数ともに増え続けるが、深刻な事態には至らない
5. 日本中に感染が広まり、死者が続出するものの、都市機能や経済に対する影響は限定的である
6. 日本中に感染が広まり、死者が続出するため、都市機能・経済が完全にマヒする

3 か月後にはどうなっていると思いますか？

6 か月後にはどうなっていると思いますか？

1 年後にはどうなっていると思いますか？

2 年後にはどうなっていると思いますか？

A9 あなたは下記のような新型コロナウイルスへの対策は適切だったと思いますか? 1 (=まったく手ぬるい・遅い)、2 (=若干手ぬるい・遅い)、3 (=とても適切・英断だった)、4 (=若干やりすぎ・拙速)、5 (=まったくやりすぎ・拙速）の 5 段階でお答えください。

1.　最近の緊急事態宣言やまん延防止等重点措置の実施に関連する政府の決定・方針 ………1　2　3　4　5

3.　ワクチン接種を日本で実施するための政府の取り組み…………1　2　3　4　5

4.　この2週間の時差出勤・テレワークの取り組み …………………1　2　3　4　5

5.　この2週間の検査・医療体制 ………………………………………1　2　3　4　5

7.　昨年実施された、国民に一律の 10 万円支給（特別定額給付金）……1　2　3　4　5

8.　収入が急減した企業・店舗への援助（持続化給付金） ………..1　2　3　4　5

9.　3 密を避ける行動の推奨………………………………………………1　2　3　4　5

A10　これから先の 1 年間(2021 年 8 月～2022 年 7 月)およびその次の 1 年間(2022 年 8 月～2023 年 7 月)に、あなたの収入や日本および世界の GDP はどのようになると予想していますか?以下に示す 1 から 7 のうち最もあなたの考えに近いものを選んでお答えください。2019 年度に比べて、1=4%以上改善する、2=1%～3.99%改善する。3=ほとんど変わらない、4=1%～3.99%下落する、5=4%～9.99%下落する、6=10%以上下落する

	これから先 1 年間<br>(2021 年 8 月～2022 年 7 月)	その次の 1 年間<br>(2022 年 8 月～2023 年 7 月)
あなたの家計の収入		
日本の GDP		
世界の GDP		

A13　以下の各項目について、あなたやあなたの家族の状況はよくなりましたか、悪くなりましたか。新型コロナ感染が始まる前の 2019 年 12 月と比較して、(1=非常によくなった、2=よくなった、3=変わらない、4=悪くなった、5=非常に悪くなった)の 5 段階でお答えください。

1.　生活の利便性…………………………………………1　2　3　4　5
2.　あなたの世帯の収入………………………………1　2　3　4　5
3.　健康状態……………………………………………1　2　3　4　5
4.　周囲の人との人間関係……………………………1　2　3　4　5
5.　仕事の状況…………………………………………1　2　3　4　5

C1　厚生労働省は、 新型コロナウイルス感染症の拡大防止に向けて、スマートフォン向けアプリ「新型コロナウイルス接触確認アプリ COCOA」を開発して、提供しています。あなたは、このアプリの存在を知っていましたか？また、このアプリをあなたのスマートフォン

に追加しましたか？

1. 既に、このアプリを自分のスマートフォンに追加した
2. 知っていて、追加するつもりである
3. 知っているが、追加するかどうかをまだ決めていない
4. 知っているが、追加するつもりはない
5. 知らなかった
6. スマートフォンを持っていない

A17

A14　あなたは以下の事項が、あなたを新型コロナウイルスから守るためにどのくらい有効だと思いますか。 1 (=全く有効でない)から 5 (=大変、有効だ)のうち当てはまるものを 1 つ選び 5 段階で答えてください。

1. 外出しない ………………………………………………….. 1 2 3 4 5
2. 職場への出社・学校への登校をしない ……………..…… 1 2 3 4 5
3. マスクの着用 ………………………………………………. 1 2 3 4 5
4. 念入りな手洗いの励行 ………………………………………. 1 2 3 4 5
5. 計画していた旅行の中止、イベント参加の見合わせ ……. 1 2 3 4 5

C2　あなたは、次の各項目で示す考え方に賛成ですか、反対ですか?
1=(完全に反対)、2(=反対)、3(=どちらとも言えない)、4(=賛成)、5(=全面的に賛成)

2. 厚生労働省や政府機関が発表する医療情報は信頼できる。
3. 日本政府を信頼している。
4. 日本の健康管理体制を信頼している。
7. ワクチンを打ったからといって外出を増やすべきではない。
8. ワクチンを打っても 2 週間は有効性は現れない

B8　1 日以内に、50%の確率で 10 万円の損失が発生するリスクがあるとします。ただし、保険料を支払っておけば、損失が発生した場合もその損失額を回収することができるものとします。あなたは保険料がいくらまでだったらこの保険をかけますか、ぎりぎり保険を掛ける値段を、下記から 1 つ選び、番号に〇をつけてください。

1. たとえ0円でも掛けない
2. 千円までなら掛ける　3. 5 千円までなら掛ける　4. 1 万円までなら掛ける

5.　1 万円 5 千円までなら掛ける　6.　2 万円までなら掛ける　7.　3 万円までなら掛ける
8.　4 万円までなら掛ける　9.　5 万円までなら掛ける　10.　5 万円以上でも掛ける

B9　1日以内に、0.1％の確率で500万円の損失が発生するリスクがあるとします。ただし、保険料を支払っておけば、損失が発生した場合もその損失額を回収することができるものとします。あなたは保険料がいくらまでだったらこの保険をかけますか、ぎりぎり保険を掛ける値段の範囲を、下記から１つ選び、番号に○をつけてください。

1.　たとえ0円でも掛けない
2.　千円までなら掛ける　3.　5千円までなら掛ける　4.　1万円までなら掛ける
5.　2万円までなら掛ける　6.　3万円までなら掛ける　7.　5万円までなら掛ける
8.　10万円までなら掛ける　9.　50万円までなら掛ける　10.　100万円までなら掛ける
11.　100万円以上でも掛ける

B10 今１万円もらうか、12 ヵ月後にいくらかもらうかのどちらかを選べるとします。今１万円もらうこと（A）と、12 ヵ月後に下記の表のそれぞれの行に指定した金額をもらうこと（B）を比較して、あなたが好む方を○で囲んでください。８つの行それぞれについて、A、または、Bを○で囲んでください。

選択肢A(円)<br>(今受取)	選択肢B(円)<br>(12ヵ月後受取)	金利(年表示)		
10,000	9,500	-5%	A	B
10,000	10,000	0%	A	B
10,000	10,200	2%	A	B
10,000	10,400	4%	A	B
10,000	10,600	6%	A	B
10,000	11,000	10%	A	B
10,000	12,000	20%	A	B
10,000	14,000	40%	A	B
